# Excerpta

**Published:** 1881-01

**Authors:** 


### EXCERPTA.

### THE EPIZOOTIC DISEASE.

Beginning a month ago, in the extreme north of New England, the so-called " epizootic disease " has extended southward, until now the horses on the Atlantic coast as far down as Washington are largely suffering from it. About forty per cent. of those in this city are at this date more or less sick. The type of disease is not severe, but it is well-known that the sequelæ are often obstinate and troublesome.

A full description of the malady is given in Tellor's *Diseases of Live Stock.* He states that the immediate cause is wholly unknown, but that the treatment is clear and efficient. The medication he recommends, from English and American authorities, is tolerably active, and careful nursing is essential. Belladonna (or atropia), he states, gives immediate relief to the spasmodic cough and noisy, difficult respiration. It were well for physicians to post themselves on this epidemic, as well for their own as for their neighbors' animals.—*Medical and Surgical Reporter.*

### SALT AS A PROPHYLACTIC IN DIPHTHERIA.

In a paper read at the Medical Society of Victoria, and published in the *Australian Medical Journal* for June, 1880, "On the Free Use of Salt as a Prophylactic against Diphtheria," Dr. Day stated that, having for many years past looked upon diphtheria in its early stage as a purely local affection, characterized by a marked tendency to take on putrefactive decomposition, he has trusted most to the free and constant applications of antiseptics; and when their employment has been adopted from the first, and has been combined with judicious alimentation, he has seldom seen blood poisoning ensue. In consequence of the great power which salt possesses in preventing the putrefactive decomposition of meat and other organic matter, Dr. Day has often prescribed for diphtheritic patients living far away from medical aid the frequent use of a gargle composed of a tablespoonful or more of salt, dissolved in a tumbler of water; giving children who cannot gargle a teaspoonful or two to drink occassionally. During the prevalence of diphtheria he recommends its use instead of sugar in the food of children, adults using the gargle as a prophylactic, three or four times a day.—*Medical and Surgical Reporter.*

DR. WILLIAM A. BYRD, of Quincy, Illinois, informs us that he believes the usual explanations which have been given for the persistent divergence of the anterior and posterior flaps, in laceration of the cervix, do not sufficiently account for the facts. In his opinion the swinging of the body of the uterus forward and backward on a point about the utero-vaginal attachment as a fulcrum, gives to the posterior lip of the lacerated cervix, as it catches on the posterior wall of the vagina, and crowds backward into the posterior vaginal cul-desac, a tendency to still greater backward rolling every time the fundus is carried backward by a full bladder or empty rectum. The anterior flap, in like manner, with a tendency to crowd forward in the axis of the vagina, has this tendency increased every time the rectum is distended, or the bladder collapsed. The idea is undoubtedly sound.—*Chicago Med. Review.*

The Apostate's Creed.—The following very clever hit at the scientific unbelief of the day, written by Mr. A. Bierbower, of this city (Cincinnati Lancet and Clinic), appears in the last issue of the New York Independent:

I believe in a chaotic nebula, self-existent, evolver of heaven and earth, and in the differentiation of the original homogenous mass, its first-forgotton product, which was self-formed into separate worlds, divided into land and water, self-organized into plants and animals, reproduced into like species, further developed into higher orders, and ultimately refined, rationalized, and perfected in man. He descended from the monkey, ascended to the philosopher, and sitteth down in the rights and customs of civilization under the laws of a developing sociology. From thence he shall come again, by the disintegration of the heterogenized cosmos back to the original homogeneousness of chaos.

I believe in the wholly impersonal absolute, the wholly uncatholic church, the disunion of the saints, the survival of the fittest, the persistence of force, the dispension of the body, and in death everlasting.—*Louisville Med. News.*

*Indolence and Longevity.*—No instance can be found of an idler having attained to a remarkably great age.

